# Recurrence due to Relapse or Reinfection With *Mycobacterium tuberculosis*: A Whole-Genome Sequencing Approach in a Large, Population-Based Cohort With a High HIV Infection Prevalence and Active Follow-up

**DOI:** 10.1093/infdis/jiu574

**Published:** 2014-10-21

**Authors:** José Afonso Guerra-Assunção, Rein M. G. J. Houben, Amelia C. Crampin, Themba Mzembe, Kim Mallard, Francesc Coll, Palwasha Khan, Louis Banda, Arthur Chiwaya, Rui P. A. Pereira, Ruth McNerney, David Harris, Julian Parkhill, Taane G. Clark, Judith R. Glynn

**Affiliations:** 1 Faculty of Epidemiology and Population Health, London School of Hygiene and Tropical Medicine; 2 Karonga Prevention Study, Malawi; 3 Faculty of Infectious and Tropical Diseases, London School of Hygiene and Tropical Medicine; 4 Wellcome Trust Sanger Institute, Hinxton, United Kingdom

**Keywords:** tuberculosis, relapse, reinfection, HIV, whole-genome sequence, RFLP, recurrence

## Abstract

***Background.*** Recurrent tuberculosis is a major health burden and may be due to relapse with the original strain or reinfection with a new strain.

***Methods.*** In a population-based study in northern Malawi, patients with tuberculosis diagnosed from 1996 to 2010 were actively followed after the end of treatment. Whole-genome sequencing with approximately 100-fold coverage was performed on all available cultures. Results of IS6110 restriction fragment-length polymorphism analyses were available for cultures performed up to 2008.

***Results.*** Based on our data, a difference of ≤10 single-nucleotide polymorphisms (SNPs) was used to define relapse, and a difference of >100 SNPs was used to define reinfection. There was no evidence of mixed infections among those classified as reinfections. Of 1471 patients, 139 had laboratory-confirmed recurrences: 55 had relapse, and 20 had reinfection; for 64 type of recurrence was unclassified. Almost all relapses occurred in the first 2 years. Human immunodeficiency virus infection was associated with reinfection but not relapse. Relapses were associated with isoniazid resistance, treatment before 2007, and lineage-3 strains. We identified several gene variants associated with relapse. Lineage-2 (Beijing) was overrepresented and lineage-1 underrepresented among the reinfecting strains (*P* = .004).

***Conclusions.*** While some of the factors determining recurrence depend on the patient and their treatment, differences in the *Mycobacterium tuberculosis* genome appear to have a role in both relapse and reinfection.

Recurrent tuberculosis adds considerably to the burden of tuberculosis [1]. It arises through relapse of the original infection or through reinfection with a new strain. The 2 mechanisms have different implications for the patient and the control program. High rates of relapse may indicate unsuccessful treatment (relapse usually occurs early after the end of treatment and is associated with cavitary disease and drug resistance) [2–4]. A high rate of recurrence due to reinfection suggests a failure to develop protective immunity after the first episode and is associated with human immunodeficiency virus (HIV) infection [2, 3, 5].

Distinguishing the 2 mechanisms requires molecular marker analysis of cultures from both episodes, so results are available from only a few studies. These have shown varying proportions of disease due to reinfection [6–8], from <3% [9] to >60% [10, 11]. High proportions of apparent reinfections may be due to mislabeling, to cross-contamination or mixed infections, or to differences in patient populations, length of follow-up [7], and background risk of infection with *Mycobacterium tuberculosis.* A high proportion of apparent relapses could indicate insufficient variability of strains in the population with the marker used. Most studies to date have used IS6110-based restriction fragment-length polymorphism (RFLP) analysis or mycobacterial interspersed repetitive unit–variable number of tandem repeat (MIRU-VNTR) typing [12].

Whole-genome sequencing gives greater discrimination than RFLP analysis or MIRU-VNTR analysis, which should improve the ability to distinguish relapse from reinfection. The one study to date that evaluated whole-genome sequencing for this purpose compared whole-genome sequencing data to MIRU-VNTR data, resulting in reclassification of several strains and allowing detection of mixed infections [12]. Whole-genome sequencing also allows genetic determinants of recurrence to be examined.

In a long-term population-based study of tuberculosis in northern Malawi with active follow-up beyond the end of treatment, we previously analyzed RFLP data from 1996 to 2005 and found that HIV infection increased the risk of recurrence due to reinfection but not the risk due to relapse [2]. Here, we have now used whole-genome sequences of all available strains obtained from patients up to 2010, and assessed the effect of HIV, *M. tuberculosis* lineage [13], and other factors on the rate of recurrence due to reinfection or relapse.

## METHODS

### Setting and Patients

The Karonga Prevention Study has been studying tuberculosis in the whole of Karonga district, northern Malawi, since 1986 [14, 15]. The area is largely rural, with a population of around 300 000 and a prevalence of HIV infection among adults of about 10%. Individuals with tuberculosis are identified through enhanced passive surveillance, with project staff based at health centers to identify those with chronic cough. Sputum and other specimens (eg, lymph node aspirates) are processed in the project laboratory. Cultures that appear to be positive for *M. tuberculosis* are sent to the United Kingdom for species confirmation and drug resistance testing, initially for resistance to isoniazid and rifampicin and, if resistant to these, then for resistance to further drugs. At least 3 sputum samples are obtained at the time of diagnosis, with further samples collected at 2 months and at the end of treatment.

Ethics approval for the study was given by the ethics committee of the London School of Hygiene and Tropical Medicine and the Malawian National Health Science Research Committee. Informed consent was sought from each participant.

The incidence of smear-positive tuberculosis in adults in this district peaked at 125 cases/100 000 in the mid 1990s and then fell to 80–90 cases/100 000 [15]. The annual risk of infection with *M. tuberculosis* in the 1980s and 1990s was estimated to be 1% per year [14]. Since 1988, HIV testing has been offered to all patients with tuberculosis, following counseling and consent; nearly two thirds are HIV positive. Free antiretroviral therapy (ART) has been available in the district since 2005 to individuals with World Health Organization stage 3 or 4 disease (which includes tuberculosis) or with CD4^+^ T-cell counts of <250 cells/mL.

Patients are treated according to Malawi government guidelines. Beginning in 1996, new smear-positive patients received a 2-month course of treatment with streptomycin, isoniazid, rifampicin, and pyrazinamide (2SHRZ) and a 6-month regimen of ethambutol and isoniazid (6HE) therapy. Ethambutol replaced streptomycin beginning in 2001. New smear-negative patients were treated with 1SEH/11HE until 2001, after which treatment was the same as that for smear-positive patients. Since January 2007, a 6-month regimen involving rifampicin throughout has been used for all new patients (2HRZE/4HR). Beginning in 1996, individuals requiring retreatment received a regimen containing rifampicin throughout [15].

Individuals were defined as having laboratory-confirmed tuberculosis if they had positive smears (excluding those with single scanty smears) or *M. tuberculosis*–positive cultures or histological evidence of tuberculosis. Outcomes beyond the end of treatment were ascertained by linkage to our other studies in the district and by home visits in 2005 to those whose vital status was unknown [16]. Since 2007 all patients have been visited annually, with sputum specimens collected from those who are symptomatic. Recurrences were defined as further episodes of diagnosed tuberculosis after the end of treatment, whether or not there was microbiological evidence of cure of the previous episode. Recurrences could be laboratory confirmed or not.

### Molecular Methods

From 1996 to 2008, IS6110 RFLP analysis was done on all cultures [17, 18]. Whole-genome sequencing was carried out on all available cultures from 1996 to 2010 (n = 1933) at the Sanger Institute, using the Illumina HiSeq 2000 system, with paired-end reads of 100 base pairs. For each of the samples, Trimmomatic software (http://www.usadellab.org/cms/?page=trimmomatic) was used to remove low-quality sequences and low-quality 3′ ends of reads, retaining only reads at least 50 base pairs long, with nucleotides above quality score Q27 (equivalent to a risk of error of <0.2% per read per base pair). The trimmed sequence reads for each sample were mapped against the H37Rv reference genome (GenBank accession no. AL123456.3) and other publicly available mycobacterial genomes, using the BWA-mem algorithm (http://bio-bwa.sourceforge.net/). Samples (n = 79) were excluded if the best match was a non-tuberculous mycobacterium or if their average genomic coverage was <10-fold. The average coverage of the remaining samples was high (median, 88-fold [range, 12–608-fold]; mean, 127-fold).

Single-nucleotide polymorphism (SNP) positions were identified using SAMtools (http://samtools.sourceforge.net/). Sample genotypes were called using the majority allele (minimum frequency, 75%) in positions supported by at least 20-fold total coverage; they were otherwise classified as missing. This strategy ignores heterozygous calls. Samples with >15% missing genotype calls were excluded, thereby removing those that may be contaminated, those containing mixed isolates, or those involving technical errors. Similarly, we excluded SNPs with >15% missing genotypes and those in highly repetitive and variable regions (eg, PE/PPE genes). In the final analysis, 94% of the *M. tuberculosis* genome was analyzed for variants. The final data set consists of 1718 samples (89% of the original cultures) and 40 046 SNPs. Raw sequence data are available from the European Nucleotide Archive (accession numbers ERP000436 and ERP001072).

Spoligotyping was performed in silico using SpolPred [19]. Lineages were defined from spoligotype families [20]. For samples with RFLP results but no whole-genome sequence data available, lineage was deduced from spoligotypes of these specimens or others with the same RFLP within the data set, as previously described [21].

### Mixed Infections

To explore whether any of the apparent reinfections were explained by the presence of a mixed infection, the number of heterozygous positions was calculated. A position was classified as heterozygous if >1 allele accounted for ≥30% of the reads (and there were >30 reads).

### Genomic Associations

To explore genomic associations with relapse, the genomes from the index episode of all individuals with molecularly confirmed relapses were compared with those from patients who were actively followed up and had no evidence of recurrence. Our in-house implementation of the *PhyC* algorithm [22] was used to explore associations showing evidence of positive selection, using a phylogenetic tree produced by RAxML [23]. Genome-wide association analysis for SNPs and annotated protein coding genes was performed using R statistical software, using logistic regression and including lineage as a covariate. For each gene, the number of mutations was aggregated by individual. A Bonferroni cutoff based on the number of independent genes with SNPs (3689) suggested a multiple testing–adjusted significance threshold of 0.000014.

### Epidemiological Analysis

Rates of laboratory-confirmed recurrence were calculated from the end of treatment to the end of June 2011. The time at risk for recurrence was censored at the date of death, leaving the district, or the first recurrence of tuberculosis, whether confirmed or not (since those receiving treatment are not at risk). HIV status was treated as a time-varying covariate for those who were HIV negative or had an unknown HIV status during the first episode. The date of seroconversion was taken as halfway between the last negative test result (or the end of the first episode if the HIV status during that episode was unknown) and the first positive test result (or the date of ART initiation if this was the first evidence of HIV positivity). Calendar time and age were also treated as time-varying covariates. Survival methods and Nelson-Aalen plots of cumulative rates were used. Rate ratios were calculated using Poisson regression, stratifying by time since the end of treatment. STATA 13 was used for all statistical analyses. Risk factors for relapse and reinfection were examined separately.

## RESULTS

### Differentiating Relapse and Reinfection

**Figure 1.**
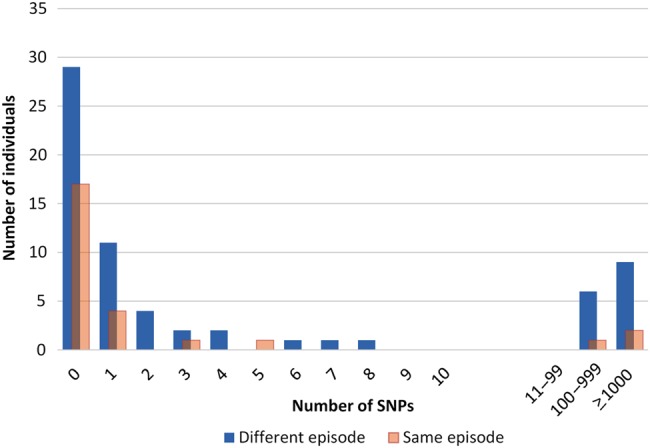
Number of single-nucleotide polymorphisms between pairs of samples from individuals from the same episode of tuberculosis or from an index episode and a recurrent episode.

After quality control, 1718 whole-genome sequences were available for analysis. These included some individuals with >1 sample from the same or different disease episode. For each of these individuals, the first and last sample were selected if there were >2 samples available. This left 92 individuals with paired samples, including 66 with samples from different episodes of disease. The number of SNPs between each of the pairs is shown in Figure 1. There is a clear gap, with pairs having a difference of ≤8 SNPs or >100 SNPs. RFLPs were available for both members of 42 pairs. All pairs (7 of 7) with a difference of >100 SNPs had different RFLPs. All pairs (35 of 35) with ≤8 SNPs different had identical RFLPs (n = 28) or RFLPs that differed by only 1 band (n = 7).

For the main analysis, only pairs from different episodes were used. Recurrence was defined as being due to relapse if there was a difference of ≤10 SNPs and as due to reinfection if there was a difference of >100 SNPs. For individuals with recurrent episodes with RFLP but no whole-genome sequencing data available, the RFLP data were used to define relapse and reinfection. Relapses had identical patterns or differed by 1 band. Reinfections had completely different patterns.

In the whole data set, the number of heterozygous positions was low for most samples, with a few having large numbers of heterozygotes. From inspection of the plot of the number of heterozygotes per sample (Supplementary Figure 1), a cutoff of 140, the inflection point in the empirical distribution of the data, was chosen to identify possible mixtures. None of the reinfections had evidence of mixed infections in either episode of disease. Two of the patients with subsequent relapse had evidence of a mixed infection in the first episode (679 and 945 heterozygous positions).

**Figure 2.**
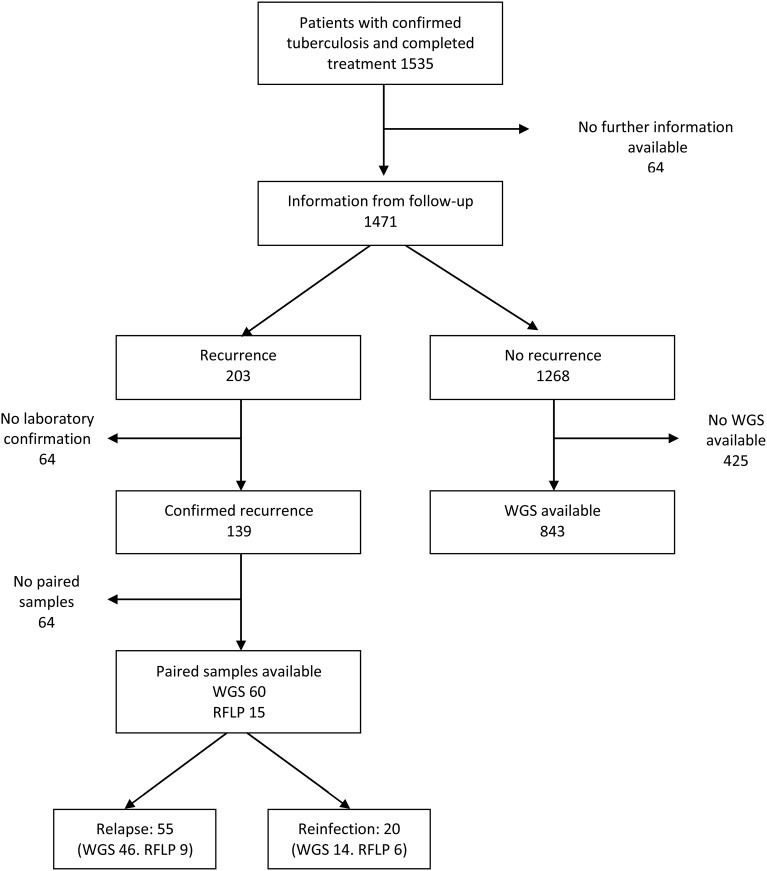
Selection of patients. Abbreviations: RFLP, restriction fragment-length polymorphism data; WGS, whole-genome sequence data.

All further analysis was restricted to individuals who received a diagnosis of laboratory-confirmed tuberculosis between January 1996 and February 2010, who were normally resident in Karonga district, and who had completed treatment. There were 1535 such individuals (Figure 2). Information beyond the end of treatment was available for 1471 (96%). Of these, 139 had laboratory-confirmed recurrences, and 64 had unconfirmed recurrences. Of the confirmed recurrences, 55 were due to relapse (defined on the basis of SNP data in 46 cases and RFLP data in 9 cases), and 20 were due to reinfection (defined on the basis of SNP data in 14 cases and RFLP data in 6 cases); 64 were unclassified (no paired samples were available). The 64 unclassified recurrences were similar to those that could be classified in terms of age, sex, HIV status, and year.

### Recurrence

**Figure 3.**
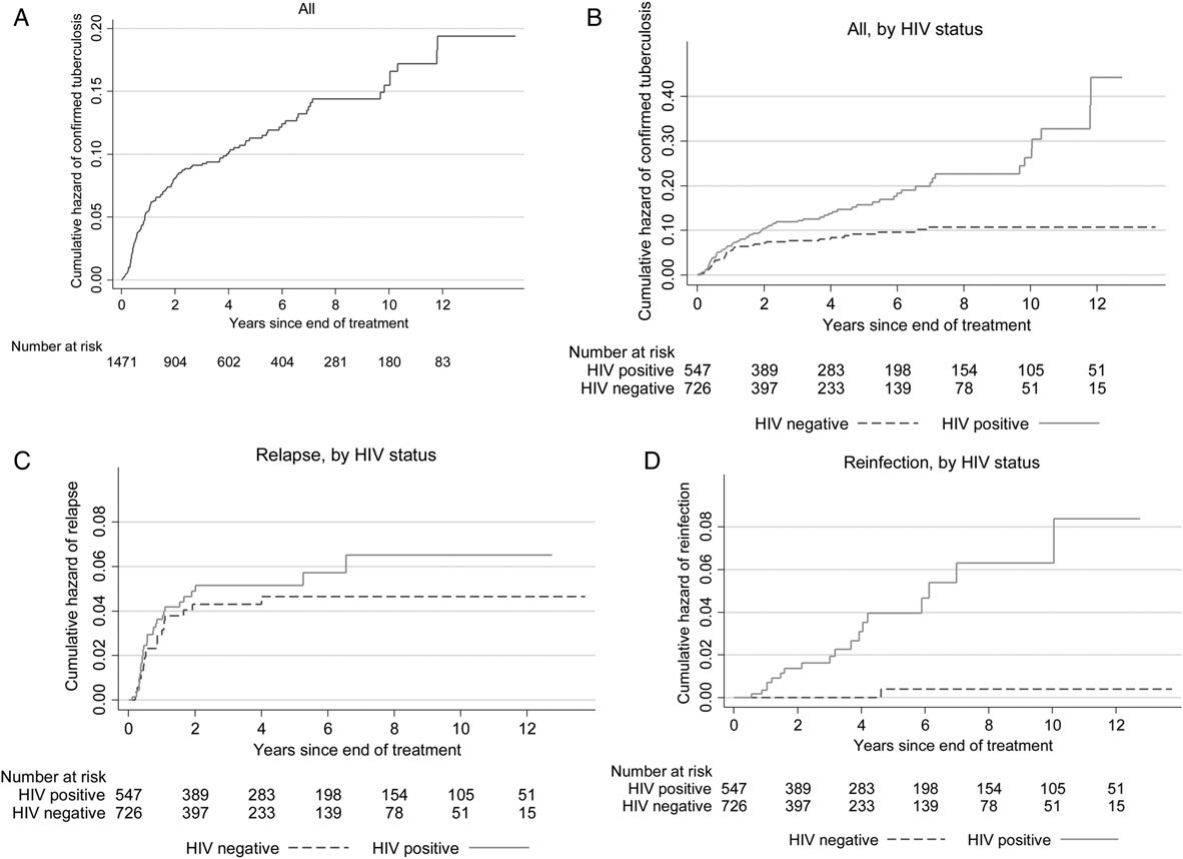
Cumulative hazard of confirmed recurrent tuberculosis overall (*A*) and by human immunodeficiency virus (HIV) status (*B*). *C* and *D*, Cumulative hazard for recurrences due to relapse and reinfection, by HIV status.

The rate of recurrence decreased by time since the end of the index episode. There were 71 recurrences in the first year, for a rate of 5.4 recurrences/100 person-years at risk (95% confidence interval [CI], 4.3–6.8); 27 in the second year, for a rate of 2.7/100 person-years at risk (95% CI, 1.8–3.9); and 41 after the second year, for a rate of 1.0/100 person-years at risk (95% CI, .76–1.4). The factors associated with recurrence within the first 2 years and beyond are shown in Table 1, and the development of recurrences, relapses, and reinfections over time are shown in Figure 3. Almost all known relapses (51 of 55) occurred in the first 2 years, whereas reinfections occurred at a more constant rate over time.

**Table 1.** Rate of Tuberculosis Recurrence, by Patient and Index Tuberculosis Episode Characteristics

Characteristic	Rate of Recurrence per 100 Person-Years at Risk					
	During the First 2 Years			After the First 2 Years		
	All Recurrences (Relapse, Reinfection)	Person-Years at Risk	Rate (95% CI)	All Recurrences (Relapse, Reinfection)	Person-Years at Risk	Rate (95% CI)
Overall	98 (51, 8)	2311	4.2 (3.5–5.2)	41 (4, 12)	3995	1.0 (.76–1.4)
Age, y						
<30	22 (13, 4)	671	3.3 (2.2–5.0)	7 (2, 0)	758	0.92 (.44–1.9)
30–39	35 (17, 2)	770	4.5 (3.3–6.3)	16 (0, 7)	1314	1.2 (.75–2.0)
40–49	19 (9, 0)	417	4.6 (2.9–7.1)	7 (1, 1)	803	0.80 (.38–1.7)
≥50	22 (12, 2)	452	4.9 (3.2–7.4)	11 (1, 4)	1051	1.0 (.58–1.9)
Sex						
Female	43 (25, 6)	1246	3.5 (2.6–4.7)	28 (1, 7)	2116	1.1 (.72–1.6)
Male	55 (26, 2)	1065	5.2 (4.0–6.7)	18 (3, 5)	1879	0.96 (.60–1.5)
HIV status						
Negative	35 (21, 0)	922	3.8 (2.7–5.3)	9 (1, 1)	1978	0.46 (.24–.87)
Positive	59 (29, 7)	1087	5.4 (4.2–7.0)	31 (3, 11)	1412	2.2 (1.5–3.1)
Unknown	4 (1, 1)	302	1.3 (.50–3.5)	1 (0, 0)	605	0.17 (.02–1.2)
Tuberculosis type						
Smear-positive pulmonary	82 (47, 4)	1894	4.3 (3.5–5.4)	35 (3, 10)	3244	1.1 (.77–1.5)
Smear-negative pulmonary	13 (3, 3)	269	4.8 (2.8–8.3)	4 (1, 1)	440	0.91 (.34–2.4)
Extrapulmonary	3 (1, 1)	148	2.0 (.65–6.3)	2 (0, 1)	310	0.65 (.16–2.6)
Previous tuberculosis						
No	90 (49, 7)	2167	4.2 (3.4–5.1)	38 (4, 11)	3759	1.0 (.74–1.4)
Yes	8 (2, 1)	144	5.5 (2.8–11.1)	3 (0, 1)	235	1.3 (.41–4.0)
*M. tuberculosis* lineage						
1	11 (6, 1)	270	4.1 (2.3–7.4)	8 (0, 3)	468	1.7 (.85–3.4)
2	1 (1, 0)	70	1.4 (.20–10.1)	1 (0, 1)	156	0.64 (.09–4.5)
3	22 (16, 0)	167	13.2 (8.7–20.0)	1 (0, 0)	212	0.47 (.07–3.4)
4	41 (27, 6)	1192	3.4 (2.5–4.7)	25 (4, 8)	2158	1.2 (.78–1.7)
Unknown	23 (1, 1)	614	3.7 (2.5–5.6)	6 (0, 0)	1000	0.60 (.27–1.3)
Isoniazid resistance						
No	76 (44, 8)	1843	4.1 (3.3–5.2)	33 (3, 9)	3344	1.0 (.70–1.4)
Yes	9 (6, 0)	81	11.1 (5.8–21.4)	4 (1, 3)	109	3.7 (1.4–9.8)
Unknown	13 (1, 0)	387	3.4 (2.0–5.8)	4 (0, 0)	542	0.74 (.28–2.0)
Year of follow-up						
1996–2000	25 (11, 6)	589	4.2 (2.9–6.3)	3 (0, 1)	151	2.0 (.64–6.2)
2001–2004	26 (19, 2)	629	4.1 (2.8–6.1)	11 (2, 4)	1042	1.1 (.58–1.9)
2005 and later	47 (21, 0)	1093	4.3 (3.2–5.7)	27 (2, 7)	2802	0.96 (.66–1.4)
Year of treatment initiation						
Before 2007	80 (47, 8)	1786	4.5 (3.6–5.6)	39 (4, 12)	3862	1.0 (.74–1.4)
2007 and later	18 (4, 0)	525	3.4 (2.2–5.4)	2 (0, 0)	132	1.5 (.38–6.0)

Abbreviations: CI, confidence interval; HIV, human immunodeficiency virus; *M. tuberculosis*, *Mycobacterium tuberculosis*.

### Relapse

Because of the very low number and rate of (confirmed) relapses after 2 years, risk factors for relapse were investigated within the first 2 years (Table 2). Relapses were more common soon after the end of treatment. They were not associated with age, sex, or HIV status. Fifty-five patients had strains that were isoniazid resistant in the index episode (including 5 that were also rifampicin resistant). Patients with isoniazid-resistant strains had 3 times the rate of relapse of those who had isoniazid-susceptible strains. Those who started treatment after 1 January 2007, who would have received rifampicin throughout their treatment, had much lower rates of relapse (rate ratio [RR], 0.29 [95% CI, .10–.80]). Those with lineage-3 strains had much higher relapse rates than those with strains from other lineages (RR, 4.2 [95% CI, 2.3–7.8], compared with lineage-4). All of these associations persisted after adjustment for each other, and additional adjustment for the other factors made no difference to the results (Table 2).

**Table 2.** Relative Rates for Relapse (Within 2 Years) and Reinfection (At Any Time)

Characteristic	Rate Ratio for Relapse Within 2 Years (95% CI)		*P* Value^b^	Rate Ratio for Reinfection Disease (95% CI)		*P* Value^b^
	Univariable	Multivariable^a^		Univariable	Multivariable^a^	
Age, y, at start						
<30	1	…		1	…	
30–39	1.1 (.55–2.3)	…		1.5 (.48–5.0)	…	
40–49	1.1 (.48–2.6)	…		0.28 (.03–2.5)	…	
≥50	1.4 (.63–3.0)	…		1.4 (.40–5.1)	…	
Sex						
Female	1	…		1	…	
Male	1.2 (.70–2.1)	…		0.61 (.25–1.5)	…	
HIV status						
Negative	1	…		1	1	<.001^c^
Positive	1.2 (.67–2.1)	…		20.9 (2.8–156)	21.0 (2.8–157)	
Unknown	0.15 (.02–1.1)	…		3.2 (.20–51.1)	2.5 (.15–40.2)	
Tuberculosis type						
Smear-positive pulmonary	1	…		1	…	
Smear-negative pulmonary	0.45 (.14–1.4)	…		2.1 (.68–6.3)	…	
Extrapulmonary	0.27 (.04–2.0)	…		1.6 (.36–6.3)	…	
Previous tuberculosis						
No	1	…		1	…	
Yes	0.61 (.15–2.5)	…		1.7 (.40–7.5)	…	
*M. tuberculosis* lineage						
1	0.99 (.41–2.4)	1.0 (.42–2.4)	.004^c^	1.3 (.43–3.9)	…	
2	0.63 (.09–4.6)	0.65 (.09–4.8)		1.1 (.14–8.0)	…	
3	4.2 (2.3–7.8)	4.2 (2.2–7.8)		…	…	
4	1	1		1	…	
Unknown	0.07 (.01–.53)	0.09 (.009–.87)		0.15 (.02–1.1)	…	
Isoniazid resistance						
No	1	1	.05^c^	1	1	.06^c^
Yes	3.1 (1.3–7.3)	2.6 (1.1–6.0)		4.8 (1.4–16.5)	4.1 (1.2–14.0)	
Unknown	0.11 (.01–.79)	0.91 (.09–9.1)		…	…	
Calendar year						
1996–2000	1	…		1	1	.001
2001–2004	1.6 (.77–3.4)	…		0.38 (.13–1.1)	0.20 (.06–.66)	
2005 and later	1.0 (.50–2.1)	…		0.19 (.07–0.54)	0.10 (.03–.32)	
Follow-up						
First year	1	1	.002	1	1	.03
Second year	0.40 (.21–.77)	0.39 (.20–.74)		3.9 (.79–19.4)	4.7 (.94–23.3)	
Third year and later	…	…		2.0 (.44–8.8)	5.9 (1.2–29.7)	
Year of treatment initiation						
Before 2007	1	…	.006	…	…	
2007 and later	0.29 (.10–.80)	0.29 (.10–.83)		…	…	

Abbreviations: CI, confidence interval; HIV, human immunodeficiency virus; *M. tuberculosis*, *Mycobacterium tuberculosis*.

^a^ Adjusted for the other factors shown in the multivariable model.

^b^ From the likelihood ratio χ^2^ test.

^c^ From the model excluding unknowns for this variable.

The genomes of isolates from the index tuberculosis episodes of 52 patients with relapse were compared with those from 843 patients with active follow-up and no recurrence of tuberculosis. An analysis of SNPs showing evidence of convergent evolution and positive selection based on the *PhyC* algorithm found 9 SNPs (from 8 genes) that were significant at the 5% level (Supplementary Table 1). Six SNPs overlapped protein-coding genes and caused nonsynonymous changes. These include the known isoniazid-resistance associated gene, *katG*. Genome-wide association analyses showed no significant genic results, after correction for multiple testing. Supplementary Table 2 lists all genes identified at a less stringent threshold (*P* < .05); this includes *katG*.

### Reinfection

The main factor associated with reinfection was HIV status (Table 2). Among HIV-negative individuals, only 1 of 44 recurrences was due to reinfection, 22 were relapses, and 21 could not be classified. Among HIV-positive individuals, 18 of 90 recurrences were due to reinfection, 32 were relapses, and 40 could not be unclassified. The one HIV-negative patient with reinfection was confirmed as being HIV negative at the time of the second episode. Reinfections were less common in more recent calendar years and were less common in the first year after the end of treatment than they were subsequently. These associations became slightly stronger after adjustment for each other, with no further change after adjustment for the other available factors. Isoniazid resistance in the first episode was also weakly associated with recurrence due to reinfection (Table 2).

Of the 20 strains causing reinfection, 1 (5%) was lineage-1, 5 (25%) were lineage-2, 2 (10%) were lineage-3, and 12 (60%) were lineage-4. This compares with a distribution of lineages of 16%, 4%, 11%, and 68%, respectively, for the 4 lineages for the index episode (*P* = .004, by the Fisher exact test). The proportion of index episodes due to lineages 1 and 2 changed little over the study, so this did not explain the difference. Of the 19 patients with reinfection for whom the lineage was known in both episodes, 10 (53%) had different lineages in the 2 episodes (compared with 48% expected by chance, given the lineage distribution in the population). For the 14 patients with reinfection defined on the basis of SNP data, the number of differing SNPs was 100–200 for 4 patients, 600–800 for 2 patients, and >1000 for 8 patients. All those with <1000 differing SNPs had lineage-4 strains in both episodes. All those with a difference of >1000 SNPs had different lineages in the 2 episodes. The one reinfection in an HIV-negative patient was with a lineage-2 strain, following lineage-1 in the initial episode.

## DISCUSSION

Whole-genome sequencing of strains from initial and recurrent episodes of disease showed a clear distinction between those with few (0–8) different SNPs and those with many (>100) differences. That this was an appropriate cutoff for distinguishing relapse and reinfection is supported by the distribution of SNP differences within the same episode (Figure 1), by the RFLP results, and by the strong correlation of relapse and reinfection so defined with time since the end of treatment and with HIV status. The cutoff is slightly higher than suggested in some other studies of SNP differences within the same episode or during transmission between patients [24–26], but there is considerable uncertainty about the appropriate cutoff, and the value will vary depending on the pipeline used and the genome coverage achieved [26]. The correlation between SNP and RFLP results suggests that an RFLP that is identical or differs by 1 band is a good approximation for defining relapse when no sequencing data are available.

In this study, none of the reinfections were explained by mixed infection. This is in contrast to findings from Cape Town, where the annual risk of infection with *M. tuberculosis* is much higher [12, 27]. We have previously found a low proportion of mixed infections in this population [28].

The timing and risk factors for relapse and reinfection in this population are strikingly different. Relapse was most common in the first year and rare after the second year. Recurrences due to reinfection were less common in the first year, but then the rate was approximately constant. HIV infection greatly increased the rate of recurrence due to reinfection but was not associated with relapse. Calendar year was also associated with recurrence due to reinfection, with higher rates before 2001, when the incidence of new tuberculosis cases was also higher [15]. Relapse was associated with isoniazid resistance (as expected [29]), treatment in earlier years (before rifampicin was given in the continuation phase, consistent with the reported effect of prolonged rifampicin on recurrence [30]), and lineage.

There is increasing interest in the extent to which genomic variation in *M. tuberculosis* may explain the variations seen in disease occurrence, patterns, and outcomes. Several studies have shown an increased rate of relapse for lineage-2 (Beijing) strains [31–33]. We did not find this, but there were only 43 patients with lineage-2 strains in the index episode, so study power was limited. We found greatly increased relapse rates for those with lineage-3 strains (East African Indian/CAS), independent of other factors. This has not been described before. In a recent study from Vietnam [33], relapse rates were compared between the Beijing strain and all other strains; lineage-3 made up about half of the comparison group. Even if all of the relapses had occurred in patients with this lineage, the rate would have been lower than the rate for those with Beijing strains. Using the *PhyC* algorithm and genome-wide association analysis, we found no genomic variants strongly associated with relapse. The expected finding of associations with *katG,* which is known to be involved in isoniazid resistance, supports the validity of the methods and suggests that the other identified genes should be investigated further for clues for mechanisms for relapse.

Although this is one of the largest studies to compare paired samples to differentiate relapse and reinfection, power is limited for genetic comparisons of this type. The lack of paired samples for 46% of the recurrences (64 of 139) will have led to underestimation of relapse and reinfection rates but is unlikely to have biased the results of associations with relapse and reinfection, as individuals without paired samples were similar to those with paired samples.

In the only previous study to use whole-genome sequencing to look at reinfection, it was suggested, based on 3 reinfections, that reinfections with strains differing by >1000 SNPs (representing different lineages) might be more likely [12]. In this analysis, 9 of 19 patients with reinfections with the lineage known for both episodes had reinfections with the same lineage, which does not support this hypothesis. However, there was some evidence that the lineage of the reinfecting strain might be important. Among episodes of recurrence due to reinfection, we found that lineage-2 strains were overrepresented and that lineage-1 (Indo-Oceanic) strains were underrepresented. This strengthens our earlier observations in this population [2] and is consistent with suggested higher virulence for lineage-2 strains [34] and lower virulence of lineage-1 strains [21]. Other studies of reinfection have not reported the results by lineage.

The use of whole-genome sequencing surmounts some of the potential problems with older techniques, namely, excluding mixed infections and knowing how much change to allow when comparing specimens from the different episodes of disease. It validated our earlier results with RFLP analysis [2] and allows the genomic associations with relapse and reinfection to be examined directly.

In this large population-based study with active follow-up, relapses were rare after the first 2 years and were associated with drug resistance and lineage-3 genotypes. Reinfections occurred at a more constant rate, were largely restricted to HIV-positive individuals, and were not more likely to be due to different lineages from the index episode but were due to lineage-2 genotypes more often than would be expected by chance.

## Supplementary Data

Supplementary materials are available at *The Journal of Infectious Diseases* online (http://jid.oxfordjournals.org). Supplementary materials consist of data provided by the author that are published to benefit the reader. The posted materials are not copyedited. The contents of all supplementary data are the sole responsibility of the authors. Questions or messages regarding errors should be addressed to the author.

Supplementary Data
