# Supplementary material for: Recurrence due to Relapse or Reinfection With Mycobacterium tuberculosis: A Whole-Genome Sequencing Approach in a Large, Population-Based Cohort With a High HIV Infection Prevalence and Active Follow-up
Source: J Infect Dis. 2014 Oct 21;211(7):1154–63. doi: 10.1093/infdis/jiu574 (PMC4354982; doi:10.1093/infdis/jiu574)
Supplement: Supplementary Data [file supp_211_7_1154__index.html]

Recurrence due to Relapse or Reinfection With Mycobacterium tuberculosis: A Whole-Genome Sequencing Approach in a Large, Population-Based Cohort With a High HIV Infection Prevalence and Active Follow-up — Recurrence due to Relapse or Reinfection With Mycobacterium tuberculosis: A Whole-Genome Sequencing Approach in a Large, Population-Based Cohort With a High HIV Infection Prevalence and Active Follow-up — Supplementary Data 

# Recurrence due to Relapse or Reinfection With *Mycobacterium tuberculosis*: A Whole-Genome Sequencing Approach in a Large, Population-Based Cohort With a High HIV Infection Prevalence and Active Follow-up

## Supplementary Data

Supplementary Data

**Files in this Data Supplement:**

- Supplementary Data - Docx file
