# Supplementary material for: Recurrence due to Relapse or Reinfection With Mycobacterium tuberculosis: A Whole-Genome Sequencing Approach in a Large, Population-Based Cohort With a High HIV Infection Prevalence and Active Follow-up
Source: J Infect Dis. 2014 Oct 21;211(7):1154–63. doi: 10.1093/infdis/jiu574 (PMC4354982; doi:10.1093/infdis/jiu574)

**Supplementary Table 1**: SNPs and genes associated with relapse from analysis based on the *PhyC* algorithm (P<0.05)

| **Genome position** | **Mutation** | ***P*** | **Gene strand** | **Gene Codon Number** | **Mutation effect** | **Gene name (Tuberculist)** | **Other gene name** |
| --- | --- | --- | --- | --- | --- | --- | --- |
| 829754 | C | 0.04 | . | . | Non-Coding |  |  |
| 874748 | T | 0.04 | + | 172 | Synonymous | [*Rv0781*](http://tuberculist.epfl.ch/quicksearch.php?gene+name=Rv0781&submit=Search) | *ptrBa* |
| 1789697 | G | 0.04 | - | 47 | NonSynonymous | [*Rv1588c*](http://tuberculist.epfl.ch/quicksearch.php?gene+name=Rv1588c&submit=Search) |  |
| 2155168 | G | 0.03 | - | 315 | NonSynonymous | [*Rv1908c*](http://tuberculist.epfl.ch/quicksearch.php?gene+name=Rv1908c&submit=Search) | *katG* |
| 2449222 | G | 0.04 | + | 355 | NonSynonymous | [*Rv2187*](http://tuberculist.epfl.ch/quicksearch.php?gene+name=Rv2187&submit=Search) | *fadD15* |
| 3253552 | A | 0.006 | + | 827 | Synonymous | [*Rv2932*](http://tuberculist.epfl.ch/quicksearch.php?gene+name=Rv2932&submit=Search) | *ppsB* |
| 3883664 | C | 0.04 | + | 47 | NonSynonymous | [*Rv3466*](http://tuberculist.epfl.ch/quicksearch.php?gene+name=Rv3466&submit=Search) |  |
| 4226372 | C | 0.04 | + | 463 | NonSynonymous | [*Rv3779*](http://tuberculist.epfl.ch/quicksearch.php?gene+name=Rv3779&submit=Search) |  |
| 4250526 | A | 0.04 | + | 217 | NonSynonymous | [*Rv3796*](http://tuberculist.epfl.ch/quicksearch.php?gene+name=Rv3796&submit=Search) | *atsH* |
|  |  |  |  |  |  |  |  |
| http://tuberculist.epfl.ch | |  |  |  |  |  |  |

**Supplementary table 2**

Genes associated with relapse from genome wide association analysis (*p*<0.05)

| Gene (Tuberculist) | OR | Lower CI | Upper CI | *p* |
| --- | --- | --- | --- | --- |
| [*htdY*](http://tuberculist.epfl.ch/quicksearch.php?gene+name=htdY&submit=Search) | 13.8 | 2.9 | 65.5 | 0.001 |
| [*Rv2877c*](http://tuberculist.epfl.ch/quicksearch.php?gene+name=Rv2877c&submit=Search) | 42.8 | 3.7 | 493.6 | 0.003 |
| [*Rv1882c*](http://tuberculist.epfl.ch/quicksearch.php?gene+name=Rv1882c&submit=Search) | 8.5 | 2.1 | 34.7 | 0.003 |
| [*sigK*](http://tuberculist.epfl.ch/quicksearch.php?gene+name=sigK&submit=Search) | 19.9 | 2.6 | 150.1 | 0.004 |
| [*pcaA*](http://tuberculist.epfl.ch/quicksearch.php?gene+name=pcaA&submit=Search) | 13.5 | 2.2 | 83.8 | 0.005 |
| [*ribD*](http://tuberculist.epfl.ch/quicksearch.php?gene+name=ribD&submit=Search) | 6.7 | 1.7 | 26.1 | 0.006 |
| [*katG*](http://tuberculist.epfl.ch/quicksearch.php?gene+name=katG&submit=Search) | 3.0 | 1.3 | 6.9 | 0.007 |
| [*Rv2454c*](http://tuberculist.epfl.ch/quicksearch.php?gene+name=Rv2454c&submit=Search) | 4.2 | 1.5 | 11.9 | 0.007 |
| [*Rv1543*](http://tuberculist.epfl.ch/quicksearch.php?gene+name=Rv1543&submit=Search) | 8.2 | 1.7 | 38.9 | 0.008 |
| [*relJ*](http://tuberculist.epfl.ch/quicksearch.php?gene+name=relJ&submit=Search) | 4.1 | 1.4 | 12.1 | 0.009 |
| [*Rv2613c*](http://tuberculist.epfl.ch/quicksearch.php?gene+name=Rv2613c&submit=Search) | 4.1 | 1.4 | 12.1 | 0.009 |
| [*Rv1888A*](http://tuberculist.epfl.ch/quicksearch.php?gene+name=Rv1888A&submit=Search) | 4.1 | 1.4 | 12.1 | 0.009 |
| [*sugC*](http://tuberculist.epfl.ch/quicksearch.php?gene+name=sugC&submit=Search) | 10.0 | 1.8 | 56.7 | 0.009 |
| [*Rv1034c*](http://tuberculist.epfl.ch/quicksearch.php?gene+name=Rv1034c&submit=Search) | 9.4 | 1.7 | 51.8 | 0.01 |
| [*echA2*](http://tuberculist.epfl.ch/quicksearch.php?gene+name=echA2&submit=Search) | 3.4 | 1.3 | 8.6 | 0.01 |
| [*lprE*](http://tuberculist.epfl.ch/quicksearch.php?gene+name=lprE&submit=Search) | 9.7 | 1.7 | 55.5 | 0.01 |
| [*Rv2742c*](http://tuberculist.epfl.ch/quicksearch.php?gene+name=Rv2742c&submit=Search) | 5.7 | 1.5 | 22.2 | 0.01 |
| [*pheS*](http://tuberculist.epfl.ch/quicksearch.php?gene+name=pheS&submit=Search) | 3.4 | 1.3 | 8.8 | 0.01 |
| [*kgtP*](http://tuberculist.epfl.ch/quicksearch.php?gene+name=kgtP&submit=Search) | 3.1 | 1.3 | 7.5 | 0.01 |
| [*Rv1954c*](http://tuberculist.epfl.ch/quicksearch.php?gene+name=Rv1954c&submit=Search) | 4.3 | 1.4 | 13.6 | 0.01 |
| [*folB*](http://tuberculist.epfl.ch/quicksearch.php?gene+name=folB&submit=Search) | 9.0 | 1.5 | 52.2 | 0.01 |
| [*Rv2017*](http://tuberculist.epfl.ch/quicksearch.php?gene+name=Rv2017&submit=Search) | 2.9 | 1.2 | 7.0 | 0.02 |
| [*tatA*](http://tuberculist.epfl.ch/quicksearch.php?gene+name=tatA&submit=Search) | 7.7 | 1.4 | 41.7 | 0.02 |
| [*lipO*](http://tuberculist.epfl.ch/quicksearch.php?gene+name=lipO&submit=Search) | 3.4 | 1.2 | 9.3 | 0.02 |
| [*rphA*](http://tuberculist.epfl.ch/quicksearch.php?gene+name=rphA&submit=Search) | 3.0 | 1.2 | 7.8 | 0.02 |
| [*udgA*](http://tuberculist.epfl.ch/quicksearch.php?gene+name=udgA&submit=Search) | 3.0 | 1.2 | 7.7 | 0.02 |
| [*Rv0585c*](http://tuberculist.epfl.ch/quicksearch.php?gene+name=Rv0585c&submit=Search) | 3.6 | 1.2 | 10.9 | 0.02 |
| [*cysA1*](http://tuberculist.epfl.ch/quicksearch.php?gene+name=cysA1&submit=Search) | 3.8 | 1.2 | 11.6 | 0.02 |
| [*Rv3168*](http://tuberculist.epfl.ch/quicksearch.php?gene+name=Rv3168&submit=Search) | 2.7 | 1.2 | 6.2 | 0.02 |
| [*pstP*](http://tuberculist.epfl.ch/quicksearch.php?gene+name=pstP&submit=Search) | 2.4 | 1.1 | 5.2 | 0.02 |
| [*murD*](http://tuberculist.epfl.ch/quicksearch.php?gene+name=murD&submit=Search) | 2.0 | 1.1 | 3.8 | 0.02 |
| [*Rv2802c*](http://tuberculist.epfl.ch/quicksearch.php?gene+name=Rv2802c&submit=Search) | 5.3 | 1.2 | 23.0 | 0.02 |
| [*esxN*](http://tuberculist.epfl.ch/quicksearch.php?gene+name=esxN&submit=Search) | 4.1 | 1.2 | 14.0 | 0.03 |
| [*dnaK*](http://tuberculist.epfl.ch/quicksearch.php?gene+name=dnaK&submit=Search) | 3.0 | 1.1 | 7.6 | 0.03 |
| [*rpsR2*](http://tuberculist.epfl.ch/quicksearch.php?gene+name=rpsR2&submit=Search) | 6.4 | 1.2 | 33.4 | 0.03 |
| [*Rv0051*](http://tuberculist.epfl.ch/quicksearch.php?gene+name=Rv0051&submit=Search) | 3.2 | 1.1 | 9.1 | 0.03 |
| [*trpB*](http://tuberculist.epfl.ch/quicksearch.php?gene+name=trpB&submit=Search) | 3.5 | 1.1 | 10.9 | 0.03 |
| [*Rv2723*](http://tuberculist.epfl.ch/quicksearch.php?gene+name=Rv2723&submit=Search) | 2.8 | 1.1 | 7.2 | 0.03 |
| [*Rv1944c*](http://tuberculist.epfl.ch/quicksearch.php?gene+name=Rv1944c&submit=Search) | 2.2 | 1.1 | 4.5 | 0.03 |
| [*rbsK*](http://tuberculist.epfl.ch/quicksearch.php?gene+name=rbsK&submit=Search) | 3.4 | 1.1 | 10.3 | 0.03 |
| [*Rv2248*](http://tuberculist.epfl.ch/quicksearch.php?gene+name=Rv2248&submit=Search) | 22.0 | 1.3 | 368.1 | 0.03 |
| [*efp*](http://tuberculist.epfl.ch/quicksearch.php?gene+name=efp&submit=Search) | 8.1 | 1.2 | 54.7 | 0.03 |
| [*Rv0692*](http://tuberculist.epfl.ch/quicksearch.php?gene+name=Rv0692&submit=Search) | 21.8 | 1.3 | 368.9 | 0.03 |
| [*Rv0997*](http://tuberculist.epfl.ch/quicksearch.php?gene+name=Rv0997&submit=Search) | 21.8 | 1.3 | 368.9 | 0.03 |
| [*Rv2513*](http://tuberculist.epfl.ch/quicksearch.php?gene+name=Rv2513&submit=Search) | 3.0 | 1.1 | 8.4 | 0.03 |
| [*vapC9*](http://tuberculist.epfl.ch/quicksearch.php?gene+name=vapC9&submit=Search) | 7.3 | 1.2 | 46.9 | 0.03 |
| [*Rv0311*](http://tuberculist.epfl.ch/quicksearch.php?gene+name=Rv0311&submit=Search) | 1.9 | 1.0 | 3.3 | 0.04 |
| [*tesA*](http://tuberculist.epfl.ch/quicksearch.php?gene+name=tesA&submit=Search) | 4.1 | 1.1 | 15.0 | 0.04 |
| [*atpG*](http://tuberculist.epfl.ch/quicksearch.php?gene+name=atpG&submit=Search) | 3.3 | 1.1 | 10.3 | 0.04 |
| [*esxC*](http://tuberculist.epfl.ch/quicksearch.php?gene+name=esxC&submit=Search) | 7.8 | 1.1 | 53.4 | 0.04 |
| [*Rv3845*](http://tuberculist.epfl.ch/quicksearch.php?gene+name=Rv3845&submit=Search) | 5.6 | 1.1 | 28.1 | 0.04 |
| [*Rv2621c*](http://tuberculist.epfl.ch/quicksearch.php?gene+name=Rv2621c&submit=Search) | 3.3 | 1.1 | 9.9 | 0.04 |
| [*Rv2314c*](http://tuberculist.epfl.ch/quicksearch.php?gene+name=Rv2314c&submit=Search) | 2.7 | 1.1 | 7.1 | 0.04 |
| [*mutB*](http://tuberculist.epfl.ch/quicksearch.php?gene+name=mutB&submit=Search) | 4.3 | 1.1 | 16.7 | 0.04 |
| [*lpqH*](http://tuberculist.epfl.ch/quicksearch.php?gene+name=lpqH&submit=Search) | 2.4 | 1.0 | 5.4 | 0.04 |
| [*recO*](http://tuberculist.epfl.ch/quicksearch.php?gene+name=recO&submit=Search) | 5.9 | 1.1 | 31.1 | 0.04 |
| [*Rv1948c*](http://tuberculist.epfl.ch/quicksearch.php?gene+name=Rv1948c&submit=Search) | 6.5 | 1.1 | 37.8 | 0.04 |
| [*Rv3000*](http://tuberculist.epfl.ch/quicksearch.php?gene+name=Rv3000&submit=Search) | 5.5 | 1.1 | 27.8 | 0.04 |
| [*fadA3*](http://tuberculist.epfl.ch/quicksearch.php?gene+name=fadA3&submit=Search) | 6.5 | 1.1 | 38.8 | 0.04 |
| [*Rv0540*](http://tuberculist.epfl.ch/quicksearch.php?gene+name=Rv0540&submit=Search) | 20.4 | 1.1 | 367.2 | 0.04 |
| [*Rv0634c*](http://tuberculist.epfl.ch/quicksearch.php?gene+name=Rv0634c&submit=Search) | 13.3 | 1.1 | 158.9 | 0.04 |
| [*Rv0828c*](http://tuberculist.epfl.ch/quicksearch.php?gene+name=Rv0828c&submit=Search) | 13.3 | 1.1 | 158.9 | 0.04 |
| [*lipJ*](http://tuberculist.epfl.ch/quicksearch.php?gene+name=lipJ&submit=Search) | 2.2 | 1.0 | 4.8 | 0.04 |
| [*ppsB*](http://tuberculist.epfl.ch/quicksearch.php?gene+name=ppsB&submit=Search) | 2.1 | 1.0 | 4.1 | 0.04 |
| [*cyp139*](http://tuberculist.epfl.ch/quicksearch.php?gene+name=cyp139&submit=Search) | 2.9 | 1.0 | 7.9 | 0.04 |
| [*Rv3405c*](http://tuberculist.epfl.ch/quicksearch.php?gene+name=Rv3405c&submit=Search) | 3.2 | 1.0 | 9.7 | 0.04 |
| [*thrC*](http://tuberculist.epfl.ch/quicksearch.php?gene+name=thrC&submit=Search) | 3.1 | 1.0 | 9.5 | 0.04 |
| [*mce2A*](http://tuberculist.epfl.ch/quicksearch.php?gene+name=mce2A&submit=Search) | 2.8 | 1.0 | 7.8 | 0.04 |
| [*uvrD2*](http://tuberculist.epfl.ch/quicksearch.php?gene+name=uvrD2&submit=Search) | 2.6 | 1.0 | 6.6 | 0.04 |

OR Odds ratio, 95% Confidence interval (CI), *p*-value

http://tuberculist.epfl.ch

Supplementary figure 1: Number of heterozygous positions per sample. Those above the line are likely to be due to mixed infections.


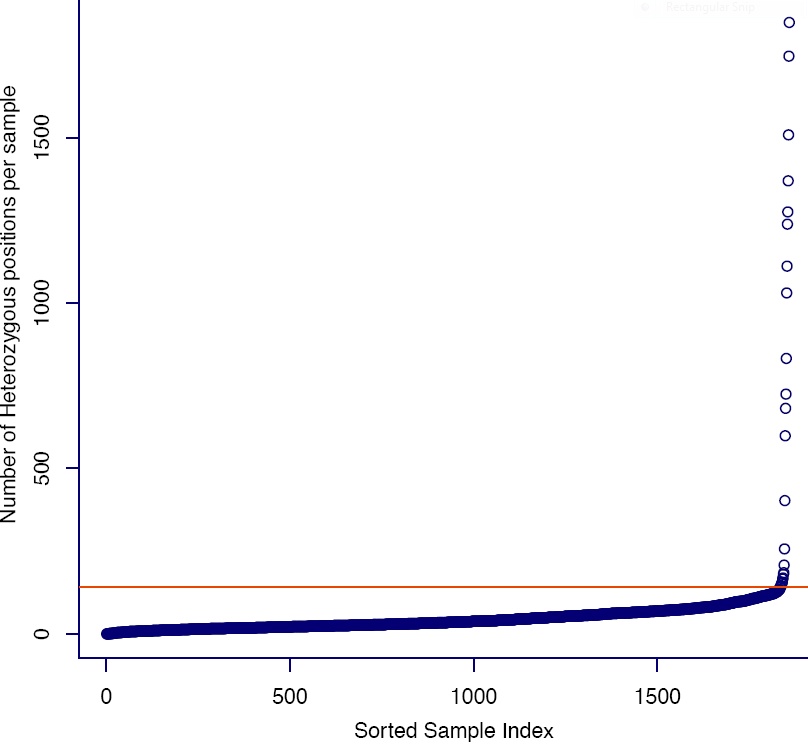

Supplement: Supplementary Data [file supp_jiu574_jiu574supp.docx]
